# Role of Microbiota in Sexually Dimorphic Immunity

**DOI:** 10.3389/fimmu.2018.01018

**Published:** 2018-05-22

**Authors:** Marlies Elderman, Paul de Vos, Marijke Faas

**Affiliations:** ^1^Division of Medical Biology, Department of Pathology and Medical Biology, University of Groningen, University Medical Centre Groningen, Groningen, Netherlands; ^2^Department of Obstetrics and Gynecology, University of Groningen, University Medical Centre Groningen, Groningen, Netherlands

**Keywords:** sex differences, microbiota, immunity, mice, humans, interfering factors

## Abstract

Sex differences in peripheral immune responses are well recognized. This is associated with sex differences in many immunological diseases. As the intestinal microbiota is known to influence the immune system, such sex differences in immune responses may be a consequence of sex-specific microbiota. Therefore, this mini-review discusses sex differences in intestinal microbiota and the possible role of microbiota in shaping sexually dimorphic immunity. Sex differences in microbiota composition are clearly found in mice studies and also in human studies. However, the lack of standardization in human studies may mask the sexual dimorphism in microbiota composition in human studies, since many factors such as age, genetic background, BMI, diet, and sex hormones appear to interfere with the sexual dimorphism in microbiota composition. Only a few mice studies found that differences in gut microbiota composition are causative for some aspects of sexually dimorphic immunity. Therefore, future studies should focus on a causal relationship between sexually dimorphic immunity and microbiota, considering the abovementioned interfering confounding factors. This would benefit the development of more sex-specific effective treatment options for immunological diseases.

## Introduction

Sex differences in immune responses are well recognized, but the mechanisms and reasons behind the dimorphic responses are still incompletely understood (1). A better insight in the reasons for differences in immune responses between the sexes might lead to more effective strategies to fight diseases in which sex plays a role, such as systemic lupus erythematosus and type 1 diabetes (T1D) (1).

**Figure 1:**
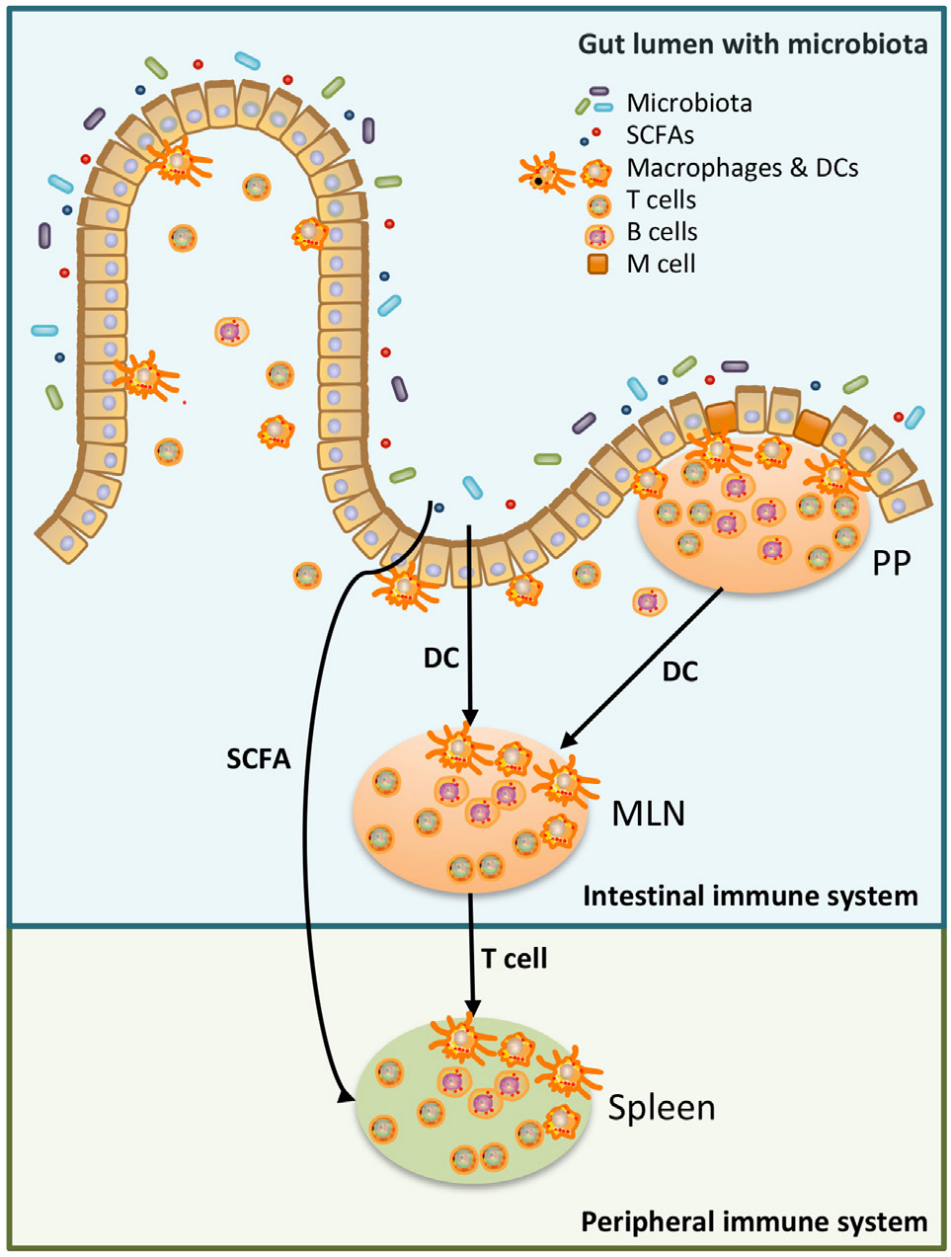
Schematic overview of the relation between microbiota, the intestinal immune system, and the peripheral immune system. Luminal microbiota and other products are continuously sampled and processed by the intestinal immune system. This could be done by specialized cells in the Peyer’s Patches (PP), microfold (M) cells, which transfer antigen to local dendritic cells (DCs). DC can present the antigen to T cells either in the PP or in the mesenteric lymph nodes (MLN). Alternatively, DC in the lamina propria sample antigen in the intestinal lumen and present antigen to T cells in the MLN (24, 25). T cells, which have recognized the antigen in the MLN, have access to the peripheral immune system and may affect the peripheral immune system (25). Moreover, metabolites produced by microbiota, such as short-chain fatty acid (SCFA), can also affect the peripheral immune system (26, 27).

Generally, both the innate immune response and adaptive immune response are stronger in females than in males (1). Recent research has shown that sex-specific differences in gut microbiota exist (2–17). Gut microbiota are in close contact with our intestinal immune system and play a major role in health and disease (18). As the immune system is partially shaped by gut microbiota this might be one of the reasons why sexual dimorphism develops. The interaction between intestinal microbes and the intestinal immune system appears to be reciprocal, since both are able to influence each other (19). Several gut bacteria, such as *Lactobacillus plantarum* and several Clostridia strains, have been shown to influence the frequency of immune cells in the intestine, such as T-regulatory cells (Tregs) (20, 21). On the other hand, the intestinal immune system is able to selectively promote the growth of specific bacteria by using mechanisms such as production of secretory immunoglobulin A (sIgA) (19). The role of sIgA in promoting specific microbiota is demonstrated by Peterson et al., who found that the production of IgA after introduction of one single bacteria into germ-free *Rag^−/−^* mice, harboring IgA-producing hybridoma cells, led to a reduced bacterial epitope expression (22). Besides influencing the intestinal immune system, gut microbiota can also shape the systemic immune system (23) (see also Figure 1).

This mini-review describes the current knowledge on sexually dimorphism in systemic and intestinal immunity and the interplay with gut microbiota. Recent exchange studies of microbiota between male and female mice have shed light on the complex relationship between microbiota, immunity, and genetics of the host. Ultimately this knowledge might lead to sex-specific strategies to manage disease.

## Sex Differences in Peripheral Immunity

Both the innate immune arm and adaptive immune arm have sex-specific differences as comprehensively reviewed by Klein et al. (1). Females were found to have a higher number of cells of the adaptive immune system such as T-helper (Th) cells and B cells (1), and a higher percentage of IL-2-producing lymphocytes (28). Males, on the other hand, were shown to have less phagocytic capacity of both macrophages and neutrophils, but a higher frequency of natural killer (NK) cells (1). Moreover, the percentage of tumor necrosis factor alpha (TNF-α), interleukin 1 beta (IL-1β), and IL-12-producing monocytes was found to be higher in males (28). These differences are held responsible for the sex bias in many immunological diseases, as the higher immune reactivity in females may contribute to their higher risk to develop autoimmune diseases, such as rheumatoid arthritis and systemic lupus erythematosus and their higher resistance to various infections as compared with males (1, 29).

Sex hormones (progesterone, estrogen, and testosterone) may play a role in the induction of sex difference in immune responses. Most immune cells in both males and females express receptors for these hormones (30), while circulating hormone levels are very different between the sexes. The impact of sex hormones on immunity is, for instance, illustrated by differences in the immune status between the follicular and the luteal phase of the ovarian cycle in women (31–33). For example, the number of peripheral Tregs is higher during the follicular phase as compared with the luteal phase (33), while the number of Th2 cells is higher in the luteal phase as compared with the follicular phase (31, 32). This is associated with low levels of progesterone and estrogen in the follicular phase and high levels of these hormones in the luteal phase (33). These temporary shifts in sex hormone levels are thought to underlie the fast and profound changes in immune status during the ovarian cycle. Also changes in the immune system after menopause in women suggest a role for sex hormones in regulating immune responses. Menopause is associated with decreased estrogen and progesterone levels (34) and has a substantial impact on the peripheral immune system, with an increase in pro-inflammatory serum markers (IL-1, IL-6, and TNF-α) and a decrease in Th cells and B cells (35).

## Sexual Dimorphism in Intestinal Immunity

Many pathogens enter the human body *via* the gastrointestinal tract. Not surprisingly the human body has developed very efficacious but specific strategies to prevent invasion of harmful invaders into the periphery (36), while at the same time the defense system should tolerate the trillions of commensal bacteria that are required for digestion of food, vitamin production (37), and production of immunological active molecules such as short-chain fatty acids (SCFAs) (38). The first line of defense is accomplished by forming a physical barrier of epithelial cells, covered by a mucus layer (36). Below and in between this barrier the body’s largest population of immune cells is located; approximately 80% of all immune cells are located here (39). The ileum contains special immune sampling sites that are not covered by mucus, which are called the Peyer’s patches (PP) (40). On top of the PP specialized epithelial cells, called microfold (M) cells, sample luminal content, which is transported to the dome of the PP for processing and recognition by the intestinal immune system (41). However, also in between the epithelial cells in the small intestine immune sampling occurs. This is done by specialized antigen-presenting cells (APCs) such as dendritic cells (DCs) and macrophages. These DCs and macrophages are present just below the surface of the epithelium and constantly sample the gut lumen. They can engulf antigens and present them to other cells, and depending on the type of antigen they either activate or attenuate an immune response (24). After encountering an antigen, DCs are known to migrate to the PP or mesenteric lymph nodes (MLN) to present their antigens to lymphocytes (36, 40). Macrophages, on the other hand, are resident and stay in the lamina propria where they have a role as intestinal innate effector cells and also communicate with other local immune cells (42). The cytokine milieu induced by luminal antigens will regulate intestinal immune cell plasticity. In case of food proteins or commensal bacteria, intestinal epithelial cells are stimulated and produce factors such as thymic stromal lymphopoietin and transforming growth factor beta (TGB-β) and retinoic acid (RA), which can condition specific CD103^+^ DCs to develop a tolerogenic phenotype (24). Besides, specific anti-inflammatory CX3CR1^+^ macrophages are able to condition CD103^+^ DCs to become tolerogenic by transferring soluble antigens to these DCs (43). Subsequently, these CD103^+^ DCs can promote the differentiation of naïve Th cells into Tregs by producing RA and TGB-β. Tregs can produce IL-10 and are important in controlling other Th responses, preventing inflammation and promoting tolerance (24, 40). However, in case of pathogens, intestinal epithelial cells and APCs will produce different factors, such as IL-1β, IL-6, and IL-23, which create a pro-inflammatory microenvironment (40, 44). This will polarize both DCs and macrophages to a pro-inflammatory phenotype (42). Subsequently, DCs will migrate to the induction sites and differentiate naïve Th cells into, e.g., Th1 or Th17 cells which will lead to inflammation and the elimination of the bacteria (44).

Despite the close interaction between the intestinal immune system and the peripheral immune system, sexual dimorphism in the intestinal immune responses is addressed in not more than a few studies (45–47). For that reason, our group studied the effect of sex on several intestinal immune cells (T cells, DCs, macrophages, and NK cells) in the PP of mice. We demonstrated that sexual dimorphism indeed also exists in the intestinal immune system and can be visualized in the PP. Males had a lower percentage of T cells, but a higher percentage of Th1 cell in the PP. Furthermore, males had a higher percentage of CD80^+^ DCs and NK cells in the PP as compared with females (45). Thus overall, male mice showed an enhanced intestinal innate immune arm and a reduced adaptive immune arm as compared with females. In another study in rats, a lower percentage of T cells was found in the MLN of males as compared with females, while also a lower percentage of macrophages was found in the male MLN as compared with the female MLN (46). In addition, many sexually dimorphically expressed genes in the small intestine and colon of mice related to immunological functions were found (47, 48).

## Sexual Dimorphism in Intestinal Microbiome

The intestinal immune system is in close contact with trillions of microbes, mainly anaerobic bacteria, together called the microbiome (49). This microbiome supports the host on various levels. Bacteria ferment dietary components, such as complex carbohydrates, that cannot be metabolized by human enzymes (37, 50). Moreover, commensal microbiota compete for luminal substrates with pathogens, preventing the growth of harmful pathogenic bacteria and thereby protect the host from pathogenic infections (37, 50). Other bacteria produce vitamins for the host, such as biotin and vitamin K, or modulate the sensitivity to hormones involved in the host energy storage, such as insulin and leptin (37, 50–52). Moreover, microbiota or microbial fermentation products, such as the SCFA butyrate, have been shown to influence immune cells (20, 21, 53). For example, some members of the microbiota, such as *Lactobacillus plantarum* and several Clostridia strains, were found to induce regulatory responses in T cells (20, 21), while others were able to stimulate specific Th17-cell responses (54). Furthermore, the SCFA butyrate has been shown to induce the differentiation of Tregs in the colon (53). The numbers and diversity of bacterial species vary along the gastrointestinal tract with a gradual increase in numbers from stomach toward the distal part of the colon (55, 56). During recent years, it has been found that a disbalance in intestinal microbiota communities may be involved in the pathogenesis of some immunological Western diseases, such as inflammatory bowel disease (IBD) and metabolic syndrome (57). Both IBD and metabolic syndrome may have a sex bias in prevalence (58, 59), which might be explained by differences in both intestinal immunity and by microbiota differences between the sexes.

It is only recently that studies demonstrated pertinent sex differences in microbiota composition (2–17) that possibly might explain differences in not only intestinal immunity but also peripheral immunity between the sexes. Most studies have been performed in rodents. Female mice were found to have an increased microbiota diversity as compared with male mice (4, 9, 17). In addition, when focusing on bacterial composition, many bacterial species were found to have a higher abundance in one of the two sexes, but in the studies performed, different bacterial species were found to be enriched in either of the sexes. For example, Sheng et al. found a higher relative abundance of *S24-7*, but a lower relative abundance of *Bacteroidaceae, Rikenellaceae, Lactobacillaceae*, and *Verrucomicrobiaceae* in male as compared with female C57BL/6 WT mice (12). Kozik et al. found that in WT B6.129S mice, males had a higher relative abundance of Ruminococcaceae, *Ruminococcus*, and *Anaerostipes*, whereas Peptostreptococcaceae was higher in females (9). These studies may indicate that sex differences in the microbiome may depend on strain, i.e., genetic background. A similar conclusion was drawn by Org et al., who investigated sex differences in gut microbiota composition in 89 different inbred strains of mice and when analyzing the strains separately, strain-specific sex differences in microbiota composition were found (5). However, this group also found that in the total cohort the phylum Actinobacteria and Tenericutes were more abundant in male than female mice. In addition, at genus level they identified *Allobaculum, Anaeroplasma*, and *Erwinia* to be more abundant in males than females, whereas *SMB53, Dorea, Coprococcus*, and *Ruminococcus* were more abundant in female mice (5).

Not only strain but also diet was found to interfere with sex effects regarding microbiota composition in mice (2, 12, 13). For instance, administration of a high fat diet to C57BL/6 mice for 81 days induced a change in microbiota composition, but the magnitude of this effect was significantly different between males and females. The high fat diet increased the abundance of several Ruminococcaceae and Lachnospiraceae, but the affected members within these bacterial families differed in males and females (13). In addition, Sheng et al. found that in C57BL/6 mice the effect of a western diet (WD), defined as a high fat and carbohydrate diet for 4 months, significantly reduced the relative abundance of *Erysipelotrichaceae* in males only, whereas this WD significantly reduced the relative abundance of *Lachnospiraceae* in females only (12).

Not only mice studies but also human studies investigated the effect of sex on the gut microbiome. Similar to the mice studies, some human studies also found sex differences in the microbiome (2, 6–8, 16), while others did not (60–62). Mueller et al. found a higher level of the *Bacteroides–Prevotella* phylogenetic group in men as compared with women (6), while Li et al. found a higher abundance of some *Clostridia, Bacteroidetes*, and *Proteobacteria* species in men as compared with women (7). Haro et al. found no significant differences in microbiota diversity, however, at the genera level they found that men had a lower relative abundance of the *Bilophila* genus and a higher relative abundance of *Veillonella* and *Methanobrevibacter* genera compared with women (16). Finally, Dominianni et al. found that men had a higher relative abundance of Bacteroidetes as compared with women (8).

**Figure 2:**
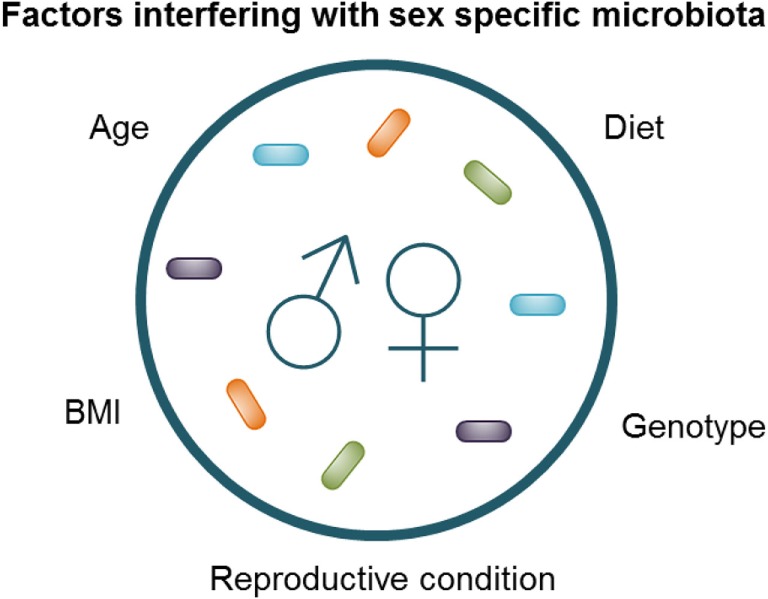
Factors interfering with sex-specific microbiota composition.

The challenge in identifying pertinent human sex differences is standardization of the studies, as it is known that factors such as age, genetic background, BMI, diet, and sex hormones can influence sexual dimorphism in microbiota (2, 4, 5, 16, 63) (Figure 2). The interaction between diet and sex was clearly shown in humans by Bolnick et al. who found that in humans the effect of diet was significantly different between men and women (2). For example, the relative abundance of *Parabacteroides* had a significant positive correlation with the amount of saturated fatty acid intake in females, while this effect was absent in males (2). In addition, the reproductive condition of females (e.g., menstrual cycle, the use of oral contraceptives and menopause), which is often not taken into account, may affect the microbiome, as effects of sex hormones on the gut microbiota have been shown by Org et al. in mice (5). Moreover, Yurkovetskiy et al. also found effects of sex hormones on the microbiota. They found that the microbiota of non-obese diabetic (NOD) mice was not different between males and females before puberty; however, after puberty male mice had a significantly less diverse microbiota, while castration of the male mice reversed these sex differences (4). Similarly, recent studies have shown that the microbiota composition changes with age (63, 64). We found that aging had a different effect in male and female mice; aged male mice had a higher abundance of *Lachnospira pectinoschiza* et rel. as compared with young male mice, while old females had a higher abundance of *Olsenella* et rel. and *Prevotella ruminicola* et rel. as compared with young females (63). Finally, Haro et al. found that men had a lower relative abundance of the *Bacteroides* genus than women, but only when their BMI was above 33 (16). The interaction between sex, immune responses, microbiota, genetic background, and environmental factors, such as diet, seemed to be in line with the results of Zelinkova and der Woude, who found that the possible sex bias in susceptibility to IBD might be related to geographical factors (e.g., genetic background and/or environmental factors) (59).

## Interaction Between Microbiome and Immune System

In view of the well-known interplay between microbiota and immunity, it seems likely that the sex differences in microbiota may be (partly) responsible for the sex differences in immune responses. To determine causal relations, Fransen et al. performed a microbiota transfer study in germ-free mice by transferring male microbiota into female germ-free mice and female microbiota into germ-free male mice (47). Fransen observed striking effects and sex-dependent differences within 4 weeks after microbiota transfer. For instance, germ-free male recipients of male microbiota had higher percentages of RORγt^+^Foxp3^+^ cells in the PPs and MLN as compared with germ-free male recipients of female microbiota. On the other hand, there were also differences that were independent of the microbiome. It was shown that males in general had higher percentages of conventional Tregs (47), irrespective of whether they received the microbiota from male or female mice. These sex differences may be caused by sex hormones or the presence of the Y or X chromosome.

Further evidence for casual relations between sexual dimorphism in microbiota and effects on immunity follows from studies in NOD mice, in which female mice have a higher chance to spontaneously develop autoimmune T1D than males. This sex bias disappears when the mice are raised under germ-free conditions, demonstrating the influence of microbiota on sex differences in development of autoimmunity and thus on sex differences in immune responses (3, 4). This was confirmed in a microbiota transfer study by Markle et al. (3). They showed that transplantation of microbiota from conventional NOD males to germ-free NOD females resulted in protection of the female mice against T1D. Since they also found this transplantation increased testosterone levels in the female mice (3), these findings suggest that both microbiota and sex hormones may be involved in the sex bias in this autoimmune disease and therefore in immune responses.

## Concluding Remarks and Future Perspectives

Present knowledge shows that sex has an effect on the microbiota composition in both mice and humans. Mice studies demonstrate that this may partly explain sexually dimorphic immunity. However, there are also effects of sex hormones and genetics on the immune system. In humans, pertinent sex differences in microbiota and effects on immunity are more difficult to proof. The absence of sex differences in several human microbiota analyses may be caused by the fact that interfering factors like reproductive condition (e.g., menstrual cycle, the use of oral contraceptives and menopause), genetic background, and diet are not taken into account but may influence the microbiota composition and immune system. A simple experiment in mice, which investigated sex differences in microbiota composition in multiple genetically distinct mice strains, revealed that sex-dependent differences were pertinent in most strains, but the specific species that differed between male and female mice were dependent on the genetic background of the strains (5). This illustrates the complexity of the interplay between microbiota, immunity, and genetics of the host and the need for highly controlled human studies in which confounding factors are as much as possible excluded. Further insight into the causal relationship between sex, microbiome, and immunity is required. This could include studies in germ-free mice, in which the effect of a single sex-specific bacterial species on the immune system could be studied. It should also include studies investigating the function of sex-specific microbiota composition and immune system, for example, in challenge models like colitis (IBD) or gastrointestinal infections, such as *Salmonella*. In these studies, the abovementioned interfering factors need to be taken into account. Such studies may result in the development of more tailored sex-specific treatment strategies. Ultimately, this knowledge might lead to sex-specific strategies to manage diseases.

## Author Contributions

All authors listed have made a substantial, direct, and intellectual contribution to the work and approved it for publication.

## Conflict of Interest Statement

The authors declare that the research was conducted in the absence of any commercial or financial relationships that could be construed as a potential conflict of interest.
